# Evidence of Combat in *Triceratops*

**DOI:** 10.1371/journal.pone.0004252

**Published:** 2009-01-28

**Authors:** Andrew A. Farke, Ewan D. S. Wolff, Darren H. Tanke

**Affiliations:** 1 Raymond M. Alf Museum of Paleontology, Claremont, California, United States of America; 2 Department of Pathobiological Sciences, School of Veterinary Medicine, University of Wisconsin-Madison, Madison, Wisconsin, United States of America; 3 Royal Tyrrell Museum of Palaeontology, Drumheller, Alberta, Canada; University of Chicago, United States of America

## Abstract

**Background:**

The horns and frill of *Triceratops* and other ceratopsids (horned dinosaurs) are interpreted variously as display structures or as weapons against conspecifics and predators. Lesions (in the form of periosteal reactive bone, healing fractures, and alleged punctures) on *Triceratops* skulls have been used as anecdotal support of intraspecific combat similar to that in modern horned and antlered animals. If ceratopsids with different cranial morphologies used their horns in such combat, this should be reflected in the rates of lesion occurrence across the skull.

**Methodology/Principal Findings:**

We used a *G*-test of independence to compare incidence rates of lesions in *Triceratops* (which possesses two large brow horns and a smaller nasal horn) and the related ceratopsid *Centrosaurus* (with a large nasal horn and small brow horns), for the nasal, jugal, squamosal, and parietal bones of the skull. The two taxa differ significantly in the occurrence of lesions on the squamosal bone of the frill (*P* = 0.002), but not in other cranial bones (*P*>0.20).

**Conclusions/Significance:**

This pattern is consistent with *Triceratops* using its horns in combat and the frill being adapted as a protective structure for this taxon. Lower pathology rates in *Centrosaurus* may indicate visual rather than physical use of cranial ornamentation in this genus, or a form of combat focused on the body rather than the head.

## Introduction

Images of the three-horned dinosaur *Triceratops* battling with conspecifics or the predator *Tyrannosaurus* have become ingrained in both the scientific and the popular mind. Lesions (wounded or diseased areas) on the horns, frill, and face of *Triceratops* specimens have been cited as evidence in support of the defensive and offensive nature of the animal's cranial ornamentation [1]–[7]. An alternative interpretation posits that these structures functioned in visual display rather than combat [1], [8]. To date, discussions of osteopathology in *Triceratops* have been anecdotal, focusing on generating speculative scenarios to explain instances of hypothesized injury [1], [7]. Without a rigorous statistical analysis, however, it is impossible to relate injury patterns to specific behaviors.

**Figure 1:**
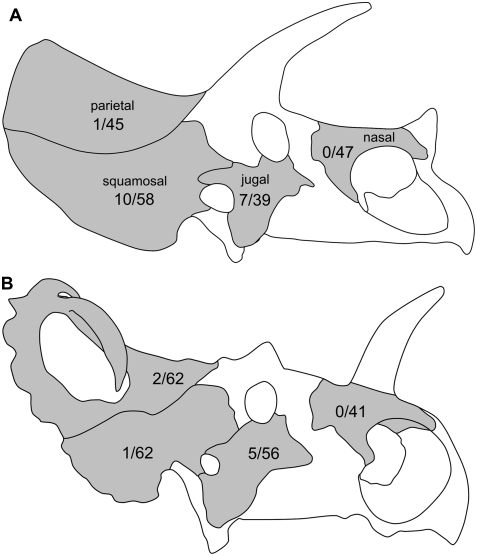
Cranial lesions in horned dinosaurs. Schematics of the skulls of (A) *Triceratops* and (B) *Centrosaurus*, showing incidence rates of lesions (periosteal reactive bone and fracture calluses) on each cranial element (number of abnormal elements / total number of elements). Not to scale.

We surveyed cranial specimens from adult individuals of the ceratopsid dinosaurs *Triceratops* and *Centrosaurus* for bony lesions (see Materials and Methods). The two animals differ greatly in cranial ornamentation; *Triceratops* has two large supraorbital horncores and a smaller nasal horncore, whereas *Centrosaurus* has a large nasal horncore and a pair of small supraorbital horncores (Figure 1). In modern horned animals, the morphology and location of the horns is closely associated with combat styles [9], [10]. By analogy, it is then expected that if *Centrosaurus* and *Triceratops* engaged in horned combat with conspecifics, the two genera would have had very different forms of combat. Thus, relative rates of lesion occurrence should differ between comparable cranial elements in both genera. If cranial ornamentations were used exclusively for visual display and/or species recognition, and not for physical contact, the two taxa are predicted to have similar rates of incidence for cranial lesions in all comparable cranial elements.

## Results

### Description of Pathologies

**Figure 2:**
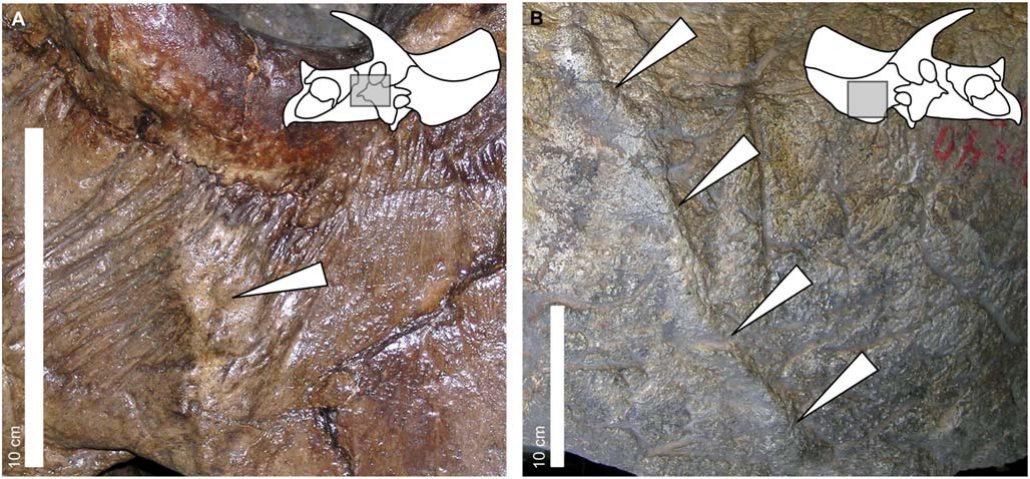
Examples of periosteal reactive bone in selected specimens of *Triceratops*. Arrows indicate lesions on (A) the left jugal of YPM 1822 (Yale Peabody Museum, New Haven, Connecticut, USA) and (B) the right squamosal of YPM 1828. The inset skull graphics indicate the approximate area of each photograph with a gray box.

**Figure 3:**
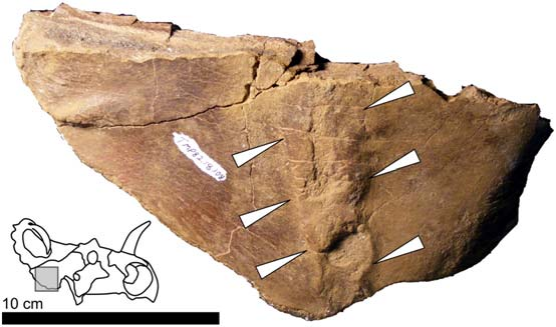
Example of a fracture callus in *Centrosaurus*. Arrows indicate a lesion on the ventral surface of the isolated right squamosal (an incomplete element) of TMP 82.18.108 (Royal Tyrrell Museum of Paleontology, Drumheller, Alberta, Canada), in ventral view. The inset skull graphic indicates the approximate area of the photograph with a gray box.

Cranial abnormalities observed in both taxa included periosteal reactive bone, healed and healing fractures, and resorptive bone lesions of unknown etiology (Figures 2,3). Only the first two categories, considered most likely due to trauma [11], were included in further statistical analysis (Figure 1, table S1).

Periosteal reactive bone reflects superficial trauma; the reaction is caused by separation of the periosteum from underlying layers and subsequent inflammatory response and healing of the bone [12]. Evidence of this injury presents as an elevated, remodeled ridge on the external surface of the bone, which may cut across the normal pattern of neurovascular impressions on the surface of the skull (Figure 2). Periosteal reactive bone was the most common of the observed pathologies (22 out of 26 observed lesions considered here).

Calluses associated with healed or healing fractures constitute the second variety of observed lesions (Figure 3; 4 out of 26 observed lesions). Such features result from the several steps of bone growth intended to reunify mechanically or pathologically separated pieces of bone. The process progresses from a primary callus with disorganized bone to a secondary callus of secondary bone [13]. Because primary bone can be preserved, calluses can be discovered in different stages of healing. The character and appearance of calluses is difficult to predict as the proliferation of bone at the site can vary from minimal to extremely exuberant depending on the individual and the severity of the fracture. In the fossil record, the fractures and calluses are often associated with an overall displacement of the bone that extends for a considerable area. Typically, fractures present as a full-thickness feature in the bone with observed disturbances of the bone fabric on both the medial and lateral aspects of the bone. This contrasts with instances of periosteal reactive bone, which affect only one side of the element.

### Statistical Analysis

A *G*-test of independence was used for all comparisons [14], [15]. *Triceratops* and *Centrosaurus* did not differ significantly in the rates of lesion occurrence within the nasal, jugal, or parietal bones of the skull (*P*>0.20 in all cases; Figure 1 and table S1 present full data). In contrast, *Triceratops* had significantly higher prevalence of lesions on the squamosal bone of the frill than did *Centrosaurus* (*P* = 0.002; see Figure 1 and table S1 for full data, and Figure 3 for the sole pathological squamosal from *Centrosaurus*).

## Discussion

We reject the possibility that a generalized pathogenic factor (such as a habitat-specific fungal infection) caused the differing prevalence of lesions between *Triceratops* and *Centrosaurus*, because all cranial elements should then show similar rates of incidence. We also rule out predatory attacks as the primary cause of the lesions, because similar large predators (tyrannosaurid theropods) were present in the habitats for both genera, and we would thus expect similar patterns of osteological abnormalities in both. Alternatively, it might be claimed that *Triceratops* had more frequent occurrence of lesions on the squamosal because this element forms a greater proportion of the frill's exposed area, and was thus more likely to be injured, than in *Centrosaurus* (e.g., Figure 1). We tested this hypothesis by comparing the prevalence of lesions in the entire frill of both taxa and still found a significant difference between the two (3 pathological and 84 non-pathological specimens for *Centrosaurus*; 10 pathological and 59 non-pathological specimens for *Triceratops*; *P* = 0.012; see Materials and Methods for full explanation). Instead, the evidence appears to be most consistent with the majority of cranial abnormalities in *Triceratops* being generated by the horns of conspecifics. The observed instances of periosteal reactive bone and healing fractures are consistent with such non-random trauma, and the elevated rates of abnormal bone morphology within the frill bones are consistent with predictions from modeling of horn-to-horn combat [2]. This suggests that the cranial ornamentation of ceratopsids, particularly *Triceratops*, was not only for visual display but that the horns also had a real role in physical combat.

It is important to note that we do not claim to infer a precise cause for individual pathologies on certain specimens (that a slip of a horn during a specific bout caused the injury to the jugal in YPM 1822, for example; Figure 2A). Certainly, at least some of the pathologies noted here may *not* be due to combat. We only claim that the *overall* pattern in all of the specimens is consistent with intraspecific combat in *Triceratops*.

Non-ceratopsid neoceratopsians (e.g., *Protoceratops*), the evolutionary predecessors of ceratopsids, possessed a thin, enlarged frill but lacked elongated brow or nasal horns. Thus, the primitive function of the frill (in addition to a role in jaw muscle attachment) was probably that of display rather than cervical protection [1]. The later evolution of brow horns would have increased the importance of a protective function for the frill, assuming that the horns were used in combat. The relatively thickened, solid frill of *Triceratops* may have been an exaptation for cervical protection, in addition to a role in display. This suggests interesting possibilities for the factors that drove the evolution of cranial morphology in ceratopsids. Display probably was an important function for the horns and frills in all ceratopsids, but not the only one. Horned combat, and the consequences of injury from this combat, may have been another important selective factor. Recent discoveries strongly suggest that *Centrosaurus* evolved from an ancestor with a *Triceratops*-like horn configuration [16]. One evolutionary interpretation worthy of further consideration is that some ceratopsids (such as *Centrosaurus*) lost their long brow horns or changed combat styles as a way to reduce cranial injury. This interpretation also suggests that the frill may not have had a protective function within *Centrosaurus* (as evidenced by the reduced occurrence of lesions on the squamosal, relative to *Triceratops*), but instead functioned for species recognition and/or other forms of visual display. *Centrosaurus* and some other ceratopsids may have focused blows on an opponent's torso rather than the skull; this is suggested by the occurrence of fractured ribs in *Centrosaurus*, *Pachyrhinosaurus*, and *Chasmosaurus* [17]. Statistical analysis and comparison with rates of rib fracture in *Triceratops*, as well as rates of cranial bony anomalies in additional taxa, may be informative in further evaluating this hypothesis. Clearly, horned dinosaurs used their cranial ornamentations for a variety of functions.

## Materials and Methods

Specimens of *Triceratops* and *Centrosaurus* were examined for evidence of bony abnormalities. In order to increase sample size, it was assumed that isolated chasmosaurine ceratopsid elements from the Hell Creek and Lance Formations were referable to *Triceratops*. This is appropriate because *Triceratops* is overwhelmingly the most common ceratopsid taxon in these formations, and because the cranial morphology (particularly the horns) is quite similar to other chasmosaurine from these formations (*Torosaurus* and *Diceratus*). No distinction was made between *Triceratops* species, *Triceratops horridus* and *Triceratops prorsus*, because of general similarity in horn morphology as well as the difficulty in assessing species for incomplete skulls. A similar approach was used for *Centrosaurus*. Two species, *Centrosaurus apertus* and *Centrosaurus brinkmani*, which differ only in minor details of the horns and frill, were combined in the sample. Additional isolated centrosaurine specimens from the Dinosaur Park Formation of Alberta were also assumed to belong to *Centrosaurus*.

Cranial elements chosen for comparison included the nasal (exclusive of the nasal horncore), jugal, squamosal, and parietal (Figure 1), and they were selected based on their abundance in the fossil record. All specimens were examined firsthand on original fossil material. Abnormalities were identified as such by comparison to “normal” elements. Individuals that were obviously juvenile or subadult (as determined by periosteal bone texture and development of ornamentation) were excluded, in order to control for possible behavioral changes during ontogeny.

Each examined element was coded as “pathological” or “normal,” and the side of the element (right or left) was also recorded. Because the parietal bone is fused into a single bilaterally symmetric element, right and left sides of the bone were distinguished relative to the midline. In *Centrosaurus*, some specimens preserved the midline bar of the frill and only one side. In this case, the elements were scored as belonging to the side that was predominantly preserved, in order to avoid inflating the sample size. Similarly, parietals consisting primarily of the midline bar were also scored only as a single element.

For each element, the data were assembled into a 2×2 matrix, with rows representing taxon and columns representing pathological state. For associated specimens preserving both right and left elements, each side was counted separately in the matrix. This is appropriate, because the pathologies counted here presumably represented discrete events in the life of the animal instead of systemic conditions [14]. In order to test the hypothesis that rates of lesion occurrence within elements were independent of taxon, a *G*-test of independence was applied to each 2×2 matrix [14], [15]. A significant test indicated that rate of occurrence was not independent of taxon.

The squamosal, relative to the parietal, forms a greater proportion of the frill in *Triceratops* than in *Centrosaurus* (Figure 1). In order to test whether this could explain why *Triceratops* more commonly exhibited lesions on the squamosal (because it could be argued that the greater surface area, rather than any behavioral factor, caused this), we conducted a second analysis looking at the frill as a single element. As before, left and right frills (one squamosal plus one half of a parietal) were counted separately, and disarticulated or isolated frill elements were considered to represent a single “frill” in order to maximize sample size. This latter assumption was modified for specimens recovered from bonebeds, in order to reduce the possibility that a single individual (represented by two squamosals and two parietals) would be over counted. Here, the number of squamosals and parietals from each bone bed sample were compared, and the element with the greatest representation was chosen as the *N* of frills for that locality. For instance, a site with 5 parietals and 8 squamosals would be considered to have 8 individual frills (rather than 13, if the numbers were just added).

## Supporting Information

Table S1 Specimens included in this study, by element. Each specimen number listed is for a single element. Where numbers are listed twice (once in pathological, once in nonpathological) or indicated with a parentheses (2), this indicates that two elements from the same individual were included in the sample. Abbreviations: AMNH, American Museum of Natural History, New York, New York; ASU, Appalachian State University, Boone, North Carolina; CCM, Carter County Museum, Ekalaka, Montana; CMN, Canadian Museum of Nature, Ottawa, Ontario; DMNH, Denver Museum of Nature and Science, Colorado; RAM, Raymond M. Alf Museum of Paleontology, Claremont, California; ROM, Royal Ontario Museum, Toronto, Ontario; SDSM, South Dakota School of Mines and Technology Museum of Geology, Rapid City; TLAM, Timber Lake Area Museum, South Dakota; TMP, Royal Tyrrell Museum of Palaeontology, Drumheller, Alberta; UCMP, University of California Museum of Paleontology, Berkeley; USNM, National Museum of Natural History, Washington, D.C.; YPM, Yale Peabody Museum of Natural History, New Haven, Connecticut. ^f^fracture callus; ^p^periosteal reactive bone
(0.02 MB DOC)

## References

[1] Farlow JO, Dodson P (1975). The behavioral significance of frill and horn morphology in ceratopsian dinosaurs.. Evolution.

[2] Farke AA (2004). Horn use in *Triceratops* (Dinosauria: Ceratopsidae): testing behavioral hypotheses using scale models. Palaeontol Electronica 7.. http://palaeo-electronica.org/2004_1/horn/horn.pdf.

[3] Lull RS (1933). A revision of the Ceratopsia or horned dinosaurs.. Mem Peabody Mus Nat Hist.

[4] Molnar RE (1977). Analogies in the evolution of combat and display structures in ornithopods and ungulates.. Evol Theory.

[5] Lull RS (1908). The cranial musculature and the origin of the frill in ceratopsian dinosaurs.. Am J Sci.

[6] Forster CA (1996). New information on the skull of *Triceratops*.. J Vert Paleont.

[7] Happ J, Larson P, Carpenter K (2008). An analysis of predator-prey behavior in a head-to-head encounter between *Tyrannosaurus rex* and *Triceratops*.. *Tyrannosaurus rex*: The Tyrant King.

[8] Horner JR, Goodwin MB (2008). Ontogeny of cranial epi-ossifications in *Triceratops*.. J Vert Paleont.

[9] Lundrigan B (1996). Morphology of horns and fighting behavior in the family Bovidae.. J Mammal.

[10] Caro TM, Graham CM, Stoner CJ, Flores MM (2003). Correlates of horn and antler shape in bovids and cervids.. Behav Ecol Sociobiol.

[11] Tanke DH, Farke AA, Carpenter K (2006). Bone resorption, bone lesions, and extra cranial fenestrae in ceratopsid dinosaurs: a preliminary assessment.. Horns and Beaks: Ceratopsian and Ornithopod Dinosaurs.

[12] McGavin MD, Zachary JF (2006). Pathologic Basis of Veterinary Disease. 4th ed.

[13] Kumar V, Fausto N, Abbas A (2004). Robbins & Cotran Pathologic Basis of Disease, Seventh Edition.

[14] Farke AA (2007). Reexamination of paleopathology in plesiosaurs.. J Vert Paleont.

[15] Sokal RR, Rohlf FJ (1995). Biometry, Third Edition.

[16] Ryan MJ (2007). A new basal centrosaurine ceratopsid from the Oldman Formation, Southeastern Alberta.. J Paleont.

[17] Tanke DH, Rothschild BM, Ryan MJ, Chinnery-Allgeier BJ, Eberth DA Paleopathologies in Albertan ceratopsids and their behavioral significance.. New Perspectives on Horned Dinosaurs.

