# Supplementary material for: Evidence of Combat in Triceratops
Source: PLoS One. 2009 Jan 28;4(1):e4252. doi: 10.1371/journal.pone.0004252 (PMC2617760; doi:10.1371/journal.pone.0004252)
Supplement: Table S1 — Specimens included in this study, by element. Each specimen number listed is for a single element. Where numbers are listed twice (once in pathological, once in nonpathological) or indicated with a parentheses (2), this indicates that two elements from the same individual were included in the sample. Abbreviations: AMNH, American Museum of Natural History, New York, New York; ASU, Appalachian State University, Boone, North Carolina; CCM, Carter County Museum, Ekalaka, Montana; CMN, Canadian Museum of Nature, Ottawa, Ontario; DMNH, Denver Museum of Nature and Science, Colorado; RAM, Raymond M. Alf Museum of Paleontology, Claremont, California; ROM, Royal Ontario Museum, Toronto, Ontario; SDSM, South Dakota School of Mines and Technology Museum of Geology, Rapid City; TLAM, Timber Lake Area Museum, South Dakota; TMP, Royal Tyrrell Museum of Palaeontology, Drumheller, Alberta; UCMP, University of California Museum of Paleontology, Berkeley; USNM, National Museum of Natural History, Washington, D.C.; YPM, Yale Peabody Museum of Natural History, New Haven, Connecticut. ffracture callus; pperiosteal reactive bone (0.02 MB DOC) [file pone.0004252.s001.doc]

| **Taxon** | **Element** | **Pathological specimens** | **Non-pathological specimens** |
| --- | --- | --- | --- |
| *Centrosaurus* | Nasal [n=41] | [n=0] | AMNH 5239 (2), 5351, 5432 (2); CMN 348 (2), 8795 (2), 8798 (2), 11837; ROM 767 (2), 12776 (2); TMP 1966.33.17 (2), 1981.22.10, 1982.18.164, 1987.18.20, 1988.18.19, 1991.36.488, 1992.36.224 (2), 1993.36.587, 1997.85.01 (2), 1998.12.69 (2), 1999.55.165, 2002.68.58, 2002.68.59, 2002.68.60, 2002.68.61, 2002.68.65, 2002.68.71; USNM 8897 (2); YPM 2015 (2); [n=41] |
| *Centrosaurus* | Jugal [n=56] | CMN 348 (2)^p^, 8795 (2)^p^; TMP 1980.18.29^p^ [n=5] | AMNH 5239 (2), 5351; CMN 1173 (2), 8797, 8798 (2), 11837; ROM 767 (2); TMP 1967.20.243, 1979.10.5 (2), 1979.11.101, 1979.11.13, 1979.11.98, 1980.18.116, 1980.18.218, 1980.18.61, 1981.18.185, 1981.18.185, 1981.18.210, 1981.18.24, 1981.18.64, 1981.18.97, 1982.18.11, 1982.18.130, 1982.18.194, 1982.18.2, 1982.18.66, 1984.18.27, 1987.18.14, 1987.18.14, 1991.18.10, 1991.18.50, 1992.36.780, 1992.36.929, 1994.12.501, 2002.68.41, 1997.85.01 (2), 2002.68.42, 2002.68.43, 2002.68.45, 2002.68.46, 2002.68.47; USNM 8897 (2); YPM 2015 (2); [n=51] |
| *Centrosaurus* | Squamosal [n=62] | TMP 1982.18.108^f^; [n=1] | AMNH 5239 (2), 5351; CMN 348 (2), 8795 (2), 8797, 8798 (2), 11837; ROM 649, 639, 767 (2); TMP 1965.12.17, 1965.12.2, 1965.12.3, 1979.10.5 (2), 1979.11.103, 1980.16.260, 1980.18.273, 1981.16.265, 1981.16.303, 1981.18.01, 1981.18.16, 1981.18.170, 1981.18.240, 1981.18.56, 1981.18.74, 1981.19.145, 1981.21.18, 1981.39.24, 1982.18.35, 1982.18.58, 1982.18.88, 1987.18.35, 1987.18.60, 1988.18.13, 1989.18.83, 1990.36.411, 1991.18.18, 1991.36.435, 1993.36.140, 1993.70.1, 1993.666.1, 1994.12.417, 1997.85.01 (2), 1998.102.6, 1999.63.22, 2002.68.112, 2002.68.113, 2002.68.114, 2002.68.20, 2005.49.137; USNM 8897 (2); YPM 2015 (2); [n=61] |
| *Centrosaurus* | Parietal [n=62] | ROM 767^f^; TMP 1995.666.69^f^; [n=2] | AMNH 5239 (2), 5351; CMN 348 (2), 8795 (2), 8798 (2); ROM 767, 793 (2); TMP 1964.5.190 (2), 1965.23.36, 1966.32.32, 1966.33.01, 1967.20.241 (2), 1967.20.247, 1979.10.5, 1979.11.109, 1979.11.18, 1980.18.26, 1980.29.104 (2), 1981.18.109, 1981.18.180, 1981.18.193, 1981.18.227, 1982.18.274, 1982.18.43, 1989.18.5, 1990.36.391 (2), 1991.18.40, 1993.40.10 (2), 1993.70.1, 1994.12.607, 1995.666.36 (2), 1996.12.281, 1997.85.01 (2), 1998.12.69, 2002.68.02 (2), 2002.68.127, 2002.68.128, 2002.68.129, 2002.68.130, 2002.68.138, 2002.68.150, 2002.68.158, 2002.68.160; USNM 8897 (2); YPM 2015 (2); [n=60] |
| *Triceratops* | Nasal [n=47] | [n=0] | AMNH 972 (2), 5116 (2); CCM 49-1, V90-11 (2); DMNH unnumbered; MOR 004 (2), 1053, 1120 (2), 1625 (2), 2923; ROM 55380 (2); SDSM 2760; UCMP 113697 (2), 137263 (2), 144297 (2); USNM 1201 (2), 1205 (2), 2100 (2), 4708 (2), 4720 (2), 5738, 8059 (2); YPM 1820 (2), 1821 (2), 1822 (2), 1823 (2), 1828; [n=47] |
| *Triceratops* | Jugal [n=39] | MOR 2923^p^; SDSM 2760^p^; UCMP 144297^p^; USNM 1205 (2)^p^, 2100^p^; YPM 1822^p^; [n=7] | AMNH 5116 (2); ASU unnumbered; CCM 49-1; CMN 8741, 8862; DMNH unnumbered; MOR 004 (2), 1053b, 1120; RAM 10017; ROM 55380 (2); TLAM unnumbered; UCMP 113697 (2), 137263, 140416,; USNM 1201, 2100, 4720, 5738, 5740, 5741; YPM 1820, 1821, 1822, 1823 (2), 1828, 2046; [n=32] |
| *Triceratops* | Squamosal [n=58] | ASU unnumbered^p^; CCM 49-1^p^; CMN 8741^p^ ; TLAM unnumbered^f^; USNM 1205 (2)^p^, 2100^p^, 5740^p^, 6525^p^; YPM 1828^p^; [n=10] | AMNH 5116 (2); ASU unnumbered; CMN 8861, 8862; DMNH unnumbered; MOR 004 (2), 1053b, 1120 (2), 1604, 1625, 2552, 2923; ROM 55380 (2); SDSM 2760; TLAM G.BA.2004.24.1; UCMP 113697 (2),137263, 140224 (2), 91MG-7-11-4; USNM 1201 (2), 2100, 2124, 2416 (2), 4286, 4720 (2), 5738 (2), 5741, 5786, 6525, 7239, “Skull 15”; YPM 1821, 1822 (2), 1823 (2), 1828, 2046; [n=48] |
| *Triceratops* | Parietal [n=45] | CCM 49-1^p^; [n=1] | AMNH 5116 (2); CMN 8741; DMNH unnumbered; MOR 004 (2), 335 (2), 1120 (2), 1186, 1604, 1625 (2), 2551 (2), 2927; ROM 55380 (2); SDSM 2760; TLAM unnumbered (2); UCMP 113697 (2); USNM 1201, 1205, 1208, 2100 (2), 2416 (2), 4720 (2), 5741 (2), 6525; YPM 1821 (2), 1822 (2), 1823 (2), 1828 (2); [n=44] |
